# Physical Activity and the Home Environment of Pre-School-Aged Children in Urban Bangladesh

**DOI:** 10.3390/ijerph18073362

**Published:** 2021-03-24

**Authors:** Jessica C. Watterworth, Jill Korsiak, Farhana K. Keya, Kelly P. Arbour-Nicitopoulos, Abdullah Al Mahmud, Vivian Tam, Daniel E. Roth

**Affiliations:** 1 Department of Nutritional Sciences, University of Toronto, Toronto, ON M5S 1A8, Canada; caymannutritionjessica@gmail.com; 2 Centre for Global Child Health, The Hospital for Sick Children, Toronto, ON M5G 2L3, Canada; jill.korsiak@mail.mcgill.ca (J.K.); viviantam6@gmail.com (V.T.); 3 International Centre for Diarrhoeal Disease Research, Bangladesh (ICDDR,B), Dhaka 1212, Bangladesh; farhana.khanam@icddrb.org (F.K.K.); mahmud@icddrb.org (A.A.M.); 4 Faculty of Kinesiology and Physical Education, University of Toronto, Toronto, ON M5S 2W6, Canada; kelly.arbour@utoronto.ca; 5 Division of Paediatric Medicine, Department of Paediatrics, The Hospital for Sick Children, 686 Bay Street, Toronto, ON M5G 1X8, Canada

**Keywords:** accelerometry, Bangladesh, children, household equipment, physical activity, sedentary behavior

## Abstract

Physical activity (PA) is a key determinant of health and development, yet few studies have examined PA levels and risk factors for low PA among young children in low- and middle-income countries. This study aimed to describe the PA and sedentary (SED) behavior levels of preschool-aged children in Dhaka, Bangladesh, and to estimate the associations between potential risk factors in the home built environment and moderate to vigorous PA (MVPA). In a sample of preschool-aged children (*n* = 65) in Dhaka, PA and SED behavior were measured for 7 days using ActiGraph GT3X-BT accelerometers. Characteristics of the home built environment, socioeconomic factors, and anthropometry were also measured. Linear mixed-effects models were used to estimate multivariable-adjusted associations between characteristics of the home environment and MVPA. Preschool-aged children spent a mean (±standard deviation) 421 ± 48 and 82 ± 23 min per day sedentary and in MVPA, respectively. There were no statistically significant associations between factors in the home built environment (indoor area, presence of an open stairwell, and presence of gross motor activity facilitating items) and MVPA. These findings suggest that the studied characteristics of the home built environment may not significantly influence the MVPA observed among preschool-aged children in Dhaka. Future research should focus on other structural and behavioral factors that facilitate PA among young children in dense urban settings.

## 1. Introduction

The double burden of malnutrition, defined as concurrent high prevalence of under- and over-nutrition, is an emerging public health issue in many low- and middle-income countries (LMICs) [1,2]. Undernutrition is a long-standing problem in LMICs, but recent changes in the built environment, food availability and access, and lifestyles have contributed to an increasing prevalence of overweight and obesity worldwide, including in lower-resource settings [2,3]. Physical activity (PA) is a key determinant of adiposity and cardiometabolic status in childhood [4] and has been shown to track from childhood to adulthood [5]; therefore, efforts to increase PA in young children are essential components of strategies to mitigate the double burden of malnutrition (under- and over-nutrition) [6,7]. Moreover, adequate levels of PA early in life may have additional benefits related to improvements in motor skills development, cognitive and social abilities, bone health, and muscle strength [4,8].

The home built environment has been hypothesized to be a determinant of PA in young children. Physical attributes of the home, such as the amount of space that is available for activity, have been found to be associated with PA in preschool-aged children [9,10,11], and the presence of PA-facilitating equipment in the home (sporting equipment, scooters and bicycles, skipping ropes, etc.) has been linked to improved motor development [12] and overall activity [9,11]. The home built environment may have a particularly prominent influence on the PA levels of preschool-aged children living in densely populated settings in LMICs. In urban Bangladesh, preschool-aged children are hypothesized to spend a considerable amount of time in the home due to limited access to communal and outdoor play spaces [13,14]. However, little is known about PA and sedentary (SED) behavior levels and their correlates among preschool-aged children in urban Bangladesh. Most previous studies investigating the association between characteristics of the home built environment and PA levels in preschool-aged children have been conducted in North America and Europe; the few studies examining PA and SED behavior among Bangladeshi children have been conducted in school-aged or older groups using subjective measures of PA, such as self- or parent report [15,16]. Among studies that employ accelerometry, approaches to quantifying PA in children vary widely; in particular, the use of different activity cut-points to define levels or categories of movement may have a substantial effect on the proportion of children deemed to meet PA targets. Yet relatively few studies have directly considered the effect of cut-point selection in studies of PA in young children.

Demonstration of the feasibility of conducting studies on PA using accelerometry in preschool-aged children in diverse settings, and new insights into the factors that affect PA among urban dwelling children in a densely populated LMIC, will help guide the development of context-relevant intervention strategies to reduce and prevent childhood overweight and obesity in these settings. The first objective of this study was to describe the PA and SED behavior levels among a sample of preschool-aged children in Dhaka, Bangladesh, to assess adherence to age-specific PA and SED behavior recommendations and to specifically consider the effect of activity-level cut-points on these estimates. The second objective was to estimate associations between characteristics of the home built environment (total area of available floor space inside the home, the number and presence of physical hazards, and the number of gross motor activity-oriented items) and children’s objectively measured daily moderate to vigorous PA (MVPA). We selected MVPA as the outcome since we assumed that the primary household factors of interest (available indoor space, physical hazards such as open stairwells, and gross motor activity-promoting toys) would be less likely to directly affect light PA (LPA) or SED behavior. The study also provided an opportunity to explore the maternal perception of the safety and suitability of the household and the surrounding environment for the child’s play.

## 2. Materials and Methods

The current study was a cross-sectional study of preschool-aged children and their mothers in Dhaka, Bangladesh. Ethical approval was obtained from the Hospital for Sick Children in Toronto, Canada (REB #1000057061), and the International Centre for Diarrhoeal Disease Research (icddr,b) in Dhaka, Bangladesh (RRC PR-17058), and all mothers provided written informed consent for participation.

### 2.1. Study Subjects

Participants were contacted and recruited from the Maternal Vitamin D for Infant Growth (MDIG) trial cohort (NCT01924013) [17]. Detailed methods and primary results of the trial have been described elsewhere [17,18]. Children between 34 and 38 months of age during the period of 17 September 2017 to 9 December 2017 who resided in Dhaka city or immediately adjacent urban communities were eligible for inclusion in this study. Participants were contacted primarily based on age at time of recruitment and excluded if they required mobility assistance (e.g., wheelchair) or were diagnosed with a major condition or disorder that substantially limits PA (e.g., cerebral palsy, clubfoot, severe asthma, or cardiac disease) (Supplementary Figure S1). In addition, participants who had since moved out of the catchment area or who did not have access to a mobile phone were not included in the study due to logistical challenges related to study follow-up (Supplementary Figure S1).

### 2.2. Data Collection

During the initial in-home visit, trained study personnel obtained maternal and child anthropometric measures and conducted an interview regarding household and socioeconomic factors, including parental education and daytime occupations, as well as got a household asset questionnaire filled out. Data collected from the household asset questionnaire were analyzed using principal component analysis to generate the first principal component. This was used to assign each individual an asset score, with lower scores reflecting ownership of fewer items (i.e., less relative wealth) and higher scores indicative of greater relative wealth.

After instructing mothers on the use of the accelerometer, each child was requested to wear the device for 7 days over a maximum 14-day period [19]. If a child was ill or had unanticipated travel away from home, only the days on which the child was healthy and at home contributed to the dataset. Daily phone calls conducted by study personnel with the mother/caregiver were used to obtain verbal reports of accelerometer wear time, child location changes, and sleep duration. At the end of the data collection period (i.e., after at least 7 completed days of accelerometry), an exit interview was conducted at the study clinic to elicit maternal report of the child’s screen time (the amount of time the child spent watching television and the amount of time spent on a screened device, such as a smartphone or a tablet), household food security status (2-item screen to identify families at risk for food security) [20], and maternal practices that were hypothesized to impact the child’s PA. Additional self-reported measures related to carrying the child indoors and outdoors, perceptions of how often the child played with other siblings or neighborhood children, perceptions of safety and cleanliness of surrounding outdoor environments, as well as the effect of these perceptions on the child’s access to these areas were also collected. In addition, the child underwent a finger-prick blood draw at the study clinic during the exit interview to measure hemoglobin. Given the high prevalence of anemia (low hemoglobin) in this setting [21] and the theoretically detrimental effect of anemia on PA [22,23,24], we considered hemoglobin to be an important descriptive characteristic of the study population and also assessed it as a potential covariate in the analyses of associations of the home built environment and PA.

### 2.3. Accelerometry

Accelerometers capture objective measurements of both PA and SED behavior and have been shown to provide robust estimates of time spent sedentary and in graded intensities of PA (i.e., light, moderate, moderate to vigorous) by young children in various settings [25,26,27]. The focus of this study was on PA, but SED behavior data were also analyzed and reported to provide a comprehensive description of the children’s activity levels throughout the waking hours of the day. PA was measured using a tri-axial GT3X-BT accelerometer (ActiGraph©, ActiGraph Corp., Pensacola, FL, USA) worn on an elastic belt around the waist. Each participant was asked to wear the device on his or her right hip over a 24 h period, only removing the device to bathe, during water activities, or if it was causing discomfort to the participant. Removal times reported by the mother during daily phone call interviews were logged in a recall report for each day the device was worn. The monitor was initialized prior to the initial home visit and began collecting data prior to the caregiver being shown how to properly position the device on the participant. The device was intended to be worn continuously for 7 days, with the option of having the child wear the device for 7 days non-consecutively within a 14-day time period if the child was traveling away from home or had an intercurrent illness (e.g., illness that the parent perceives as greatly affecting PA, such as fever or a major injury). Valid wear time was defined as at least 3 days, with a minimum of 10 h of daytime wear per day [19].

### 2.4. Anthropometry

Child and maternal heights and weights were measured to the nearest 0.1 cm and 0.01 kg, respectively, taken in duplicate and averaged. Child height was measured using a wooden length board (ShorrBoard^TM^, Shorr Productions, Olney, MD, USA) and maternal height using a stadiometer (Seca-217, Seca Corp., Hamburg, Germany). Weight was measured using a portable scale (Seca-874, Seca Corp., Hamburg, Germany). Standardized age- and sex-specific z-scores for height and weight were calculated based on the World Health Organization (WHO) growth standards for all child participants [28]. Maternal body mass index (BMI) was calculated and categorized using the standard WHO adult cut-offs (<18.5 kg/m^2^ indicated underweight, 18.5–24.9 kg/m^2^ indicated normal weight, 25–29.9 kg/m^2^ indicated overweight, ≥30 kg/m^2^ indicated obesity) [29]. For descriptive purposes, waist circumference and bioelectrical impedance analysis (BIA) were measured for both mother and child. Waist circumference was measured at the top of the iliac crest after a normal exhalation using a non-stretch tape measure (Gulick II measuring tape, Country Technology Inc., Gays Mills, WI, USA). BIA was measured with the participant laying supine, using the hand-to-foot technique, whereby the resistance and reactance of an electrical current is measured through the body from the right hand to the right foot, finding the path of least resistance (Quantum II—Body Composition Analyzer^TM^; RJL Systems, Clinton Township, MI, USA). Child and maternal body fat percentages were calculated using validated age- and population-appropriate equations [30,31,32].

### 2.5. Home Built Environment

During the initial home visit, each caregiver was asked where his or her child spent the majority of the time and this location was identified as the primary residence. A home audit was conducted by study personnel to evaluate the built environment of the participant’s primary residence. The caregiver was asked to identify any restricted areas within the home to which the child was not allowed access. Then, the study staff constructed a schematic diagram of the indoor space available to the child, including any furniture that was greater than 1 m^2^ (lengths and widths were measured using a standard tape measure), and measured the dimensions of the indoor rooms using a laser measuring device (Disto^TM^ D1, Leica Geosystems, Switzerland). All measurements were taken in duplicate and averaged. Beds and sofas were not included in the diagram, as it was observed that they were often used as a play space by children; so the area they occupied was included in the available play space. With consent, photographs of each room were taken to accompany room measurements and schematic drawings to improve the accuracy and objectivity of the home audit. The indoor area (m^2^) available to the child was calculated by subtracting the area occupied by the furniture (except beds and sofas) from the total interior room area. Study personnel then assessed the home for the following common physical hazards in urban Dhaka: an open stairwell (no railing present), exposed sewage drains (accessible to the child), unfinished construction within the home (accessible to the child), accessibility to open flames for cooking, and open storage of a *boti* (a large household knife commonly used in their daily practices by Bangladeshi families). These hazards were identified *a priori*, based on staff observations collected during home visits during the MDIG trial. Due to difficulties in objectively evaluating the presence of unfinished construction, as well as homogeneity of the sample with regard to access to open flames and open storage of a *boti*, the latter three hazards were excluded from our analyses. Finally, study personnel took an inventory of gross motor and fine motor activity facilitating items in the home by asking the mother to identify play items in the home to which the child had access. Gross motor activity items are defined as household items or play equipment that encourage active whole-body motion (e.g., walking, running, jumping, climbing). Photographs of each item were taken, and each item was independently classified by two assessors as a fine motor activity item, a stationary/fixed gross motor activity item, or a portable gross motor activity item (fixed equipment, ball play equipment, push/pull equipment, locomotor equipment, rocking and twisting equipment), based on the Affordances in the Home Environment for Motor Development (AHEMD) [33] and the Environment and Policy Assessment and Observation (EPAO) [34] home and daycare equipment inventory questionnaires. Only items classified as gross motor activity items were included in analyses.

### 2.6. Hemoglobin Concentration

Blood samples were obtained by pricking the left index finger with a spring-loaded needle with the child in a seated position. The first drop of blood was wiped away and the second drop was used to obtain a hemoglobin concentration (g/L) (HemoCue^®^, Helsingborg, Sweden). Anemia was defined as a hemoglobin concentration less than 110 g/L.

### 2.7. Statistical Analyses

Data from paper data forms were double-entered independently using the REDCap^®^ (Research Electronic Data Capture) platform [35]. Any discrepancies between data enterers were flagged and reconciled. Normality of continuous variables was assessed visually using histograms, normal quantile–quantile (Q-Q) plots, and box plots. Descriptive statistics included means and standard deviations (SDs), medians and interquartile ranges (IQR), and frequencies and percentages, where appropriate. The ActiGraph© GT3X-BT was set to collect data at a sampling rate of 30 samples per second, and activity data were integrated into 15 s intervals (epochs) to accommodate the sporadic movement patterns observed in young children [36,37]. Data were analyzed using activity counts in the vertical axis (the axis that captures movement in the up–down direction and most often used to indicate natural movement) as this was the only axis in which cut-points have been previously calibrated and validated in young children [38]. Detailed accelerometer parameters applied in this study are summarized in Supplementary Table S1. The primary measure of PA was a continuous variable reflecting mean daily PA during the study period, expressed as mean counts per 15 s (cp15s). This summary measure was generated by first calculating mean daily PA, then averaging the daily means across collection days, including data between the time the child woke up and the time the child went to sleep, as reported by the caregiver on all days that met the criteria for inclusion (i.e., at least 10 h of wear time) [19]. Due to the young age of the participants, we expected that compliance for a full 7 days might be difficult; therefore, prior to undertaking the statistical analyses, we decided to use 3 days of data as the minimum number of valid days per child to include a child in the analyses [39]. If a child had more than 7 days of eligible wear time, we included all days with sufficient wear time. We excluded non-wear periods when the accelerometer was not being worn by the child (based on daily logs collected from the caregivers via afternoon telephone calls). We also excluded non-wear periods defined by the software program (ActiLife version 10; ActiGraph Corp., Pensacola, FL, USA), using custom settings recommended for accelerometry analyses [40]. Non-wear time was defined as 20 min or more of consecutive zero counts in the vertical axis (the axis that measures up–down movement, most commonly cited in the literature), with a maximum of 20,000 counts per minute and a spike tolerance of 2 min [41]. Any wear periods that fit this definition were excluded from the total wear time. The 15 s epochs were used to calculate the time (and percentage of total time) spent in each PA and SED category; in primary analyses, we used activity cut-points established by Trost et al. (SED: ≤48 counts per 15 s (cp15s); light PA (LPA): 49–418 cp15s; and moderate to vigorous PA (MVPA): >418 cp15s) [38]. As there are currently no national PA guidelines for preschool-aged children in Bangladesh, PA targets were determined by comparing sample activity means to the WHO Guidelines on physical activity, sedentary behavior and sleep for children under 5 years of age [42], which are comparable to many existing national movement guidelines [43]. The WHO Guidelines provide the following daily recommendations for three movement behavior categories for children 3–4 years of age: PA (at least 180 min of total physical activity (TPA), of which at least 60 min is energetic play or MVPA), sedentary screen time (no more than 1 h per day), and sleep (10–13 h of good-quality sleep). To assess the sensitivity of the descriptive analyses to cut-point criteria, we repeated the analyses by applying three other cut-points developed for preschoolers (Pate et al. [44], Sirard et al. [45], and van Cauwenberghe et al. [46]) (Supplementary Table S2). The intraclass correlation coefficient (ICC) from an intercept-only linear mixed model was used to partition the within- versus between-participant variance in average daily total PA.

To address the second objective, we used linear mixed-effects models with child-specific random intercepts to estimate associations between each household factor and average daily minutes of MVPA. Models were adjusted for the following covariates considered *a priori* to be possible confounders based on directed acyclic graphs specific to each individual model: child gender, household density (people/m^2^), food security status (food secure vs. insecure), maternal and paternal education, and household wealth (asset index score). Given the null association between child hemoglobin concentration and PA level (Supplementary Table S3 and Supplementary Figure S2), hemoglobin was not considered further as a covariate in analyses. In sensitivity analyses, we excluded parent-reported sick days from the data (Supplementary Table S4).

*p*-Values < 0.05 were considered statistically significant. All statistical analyses were performed using STATA version 13.1 (College Station, TX, USA).

## 3. Results

### 3.1. Participant Characteristics

The enrolled study sample consisted of 37 (57%) boys and 28 (43%) girls, with a mean age of 37 months (approximately 3 years) and low-average-weight and height-for-age z-scores in the children but a high prevalence of overweight/obesity in the mothers (Table 1; Supplementary Figure S1). The median available indoor area was 18.2 m^2^ (IQR 17.8 m^2^). Most mothers (88%) reported children had access to outdoor play spaces (e.g., balcony, rooftop, nearby greenspace). Home measures (length and indoor area) had high inter- and intra-rater reliability, with correlation coefficients of 0.999 and 0.998, respectively (Supplementary Tables S5 and S6). All participants met minimum-wear-time criteria (at least 3 valid days of 10 h of wear time), thereby contributing to 100% accelerometer wear compliance (Supplementary Table S7). Mothers reported that accelerometer removal was mostly during bathing, with three participants reporting device removal at night due to discomfort. All participants contributed at least 2 weekdays and 1 weekend day of accelerometry data.

### 3.2. Physical Activity Levels

The overall mean (±SD) average daily count in the vertical axis was 140 ± 25 cp15s. On average, time was approximately evenly divided between SED behavior and PA (including LPA and MVPA) and did not markedly vary by participant gender, food security status, or day of the week (Table 2). Similar results were obtained when removing parent-reported sick days (seven observations) from the data (data not shown). A substantial proportion of the overall variance in PA was explained by day-to-day variability within each child (ICC = 0.38; Supplementary Figure S3).

Applying the WHO Movement Guidelines, most children (84%) met PA recommendations of at least 180 min per day of TPA and 60 min per day of MVPA. All participants achieved at least 30 min per day of MVPA, and 94% achieved at least 45 min of MVPA. Parent-reported screen time revealed that 34% of the sample received less than 1 h per day of television viewing and 22% received less than 1 h per day of screen time from portable devices (smartphone or tablet). A low proportion of mothers (9%) reported that their children did not have any screen time. Of the sample, 34% met sleep recommendations of 10–13 h of sleep each night. The proportions of the sample that met combinations of PA, SED screen time, and sleep category recommendations were 34% for PA and SED behavior, 35% for PA and sleep, and 12% for SED behavior and sleep. Only 12% of the participants met recommendations for all three categories.

The mean proportion of valid days for which each participant met PA recommendations of at least 180 min per day of TPA was 99% (±2 SD). On average, participants had a large proportion of valid days with at least 30 min of MVPA (98 ± 6%), but the proportion decreased as the number of minutes per day of MVPA increased such that the average proportions of days for which a participant achieved at least 45 and 60 min per day of MVPA were 90 ± 2% and 74 ± 3%, respectively. The average proportion of days for which each participant achieved 10–13 h of sleep was 42 ± 30%.

In sensitivity analyses, use of three alternative age- and device-appropriate activity cut-points calibrated and validated in similar age groups [44,45,46] led to discrepancies in PA classification, particularly when comparing time spent SED and in MVPA and TPA (Supplementary Table S8). Average SED time ranged from 52% of daily wear time (Trost cut-points) to 88% (van Cauwenberghe). Mean daily MVPA ranged from 5% of wear time (Sirard) to 10% (Trost). Discrepancies in the proportion of the sample that met PA category recommendations of the WHO Guidelines were also observed across the four cut-points. The proportion of participants who achieved 180 min per day of TPA ranged from 0% (van Cauwenberghe) to 100% (Trost), and the proportion that achieved 60 min per day of MVPA ranged from 8% (Sirard) to 84% (Trost).

### 3.3. Association of Household Factors with Physical Activity

Available indoor area (m^2^) was positively associated with preschool-aged children’s average daily MVPA, but the association was attenuated and no longer statistically significant in the multivariable model that included household density, asset index score, maternal and paternal education category, and food security status as covariates (Table 3). Other household factors were not significantly associated with average daily MVPA. In sensitivity analyses, the exclusion of parent-reported sick days did not substantially affect the effect estimates (Supplementary Table S4). In post-hoc exploratory analyses, we found that boys (versus girls), higher asset index (a proxy of household socioeconomic status), and higher maternal education were associated with higher average MVPA (Supplementary Table S9).

### 3.4. Maternal Perceptions of the Home and the Surrounding Environment

A majority of mothers perceived their homes as having adequate indoor space to facilitate PA (65%) and did not feel comfortable allowing their children to play outdoors (78%; Table 1). When asked to provide reasons they would prevent their children from playing outdoors, the most common concerns related to lack of safety (82%) or cleanliness (55%) and inadequate outdoor space (43%) (Table 1). A majority of mothers (80%) reported that their children played with siblings or neighborhood friends (Table 1). Mothers also reported that their children were frequently carried when indoors and outdoors (Table 1).

## 4. Discussion

In this study in a dense urban community in Dhaka, Bangladesh, the majority of preschool-aged children met PA recommendations (based on WHO Movement Guidelines), achieving at least 180 min per day of PA of any intensity and 60 min per day of MVPA. We did not find statistically significant associations between home environment characteristics and children’s average daily MVPA. However, this study demonstrated high device wear compliance and multiple days of suitable activity data generated in all participants, thereby supporting the feasibility and acceptability of using accelerometers to quantify PA and SED behavior in this population in future research.

Recent efforts to summarize and compare PA levels in children and youth worldwide have been limited to school-aged children and adolescents (5 to 17 years) due to the recognized lack of PA data for children under age 5 years [47]. We did not identify other studies of preschoolers in Bangladesh or other South Asian countries to which the present results may be directly compared. In two previous studies conducted in densely populated urban centers in China, the majority of young children were found to meet MVPA recommendations [48,49]. In contrast, the average preschooler TPA and MVPA levels were lower in preschoolers studied in Singapore and Japan [50,51]. Beyond the variability in cultural and socioeconomic factors that might affect preschooler PA levels, differences in methods of PA data collection and analysis (e.g., accelerometer device chosen, wear location of the device, cut-points) limit direct comparisons among such studies. Objectively measured PA data among preschool-aged children in LMICs are urgently needed to guide public health action; ideally, such data should be generated using uniform approaches to PA data collection and analysis to enable meaningful between-country comparisons and to track progress over time. The present study highlighted the particular importance of establishing standardized cut-points when classifying activity levels.

Contrary to our hypothesis, available indoor area was not associated with preschool-aged children’s average daily MVPA after adjustment for hypothesized confounders. The observed effect size was small, so we did not attribute the null finding to a lack of statistical power/precision. This finding is inconsistent with prior studies in North America and Europe, in which the indoor areas of homes and home-based childhood settings positively correlated with MVPA levels in young children [9,10,11]. Furthermore, a study in urban Brazil found that preschools that allowed children access to indoor recreation rooms or park spaces, regardless of the size of the space, protected against high levels of SED behavior [52]. However, a potential limitation of these studies was that the indoor area (or the presence of an indoor play space) was measured using questionnaires. Although such tools have been validated in this age group, caregiver- or researcher-reported measures may be subject to bias and measurement error. In the present study, we quantified available indoor area using a laser measuring device, a method that has not been used previously to determine the amount of space available to children for active play. Consistent with our original hypothesis, most mothers did not feel that the surrounding outdoor spaces were suitable for the children’s active play. However, the majority of mothers perceived their homes as having adequate space for their children to move freely, which may partly explain the lack of an association between indoor space and children’s MVPA.

Some household hazards frequently encountered in this study (e.g., stairwells without railings or barriers) are less common in high-income countries and have not been extensively studied previously, and their near ubiquity in this population likely contributed to the lack of observed associations with MVPA. Results of previous studies on the association between play equipment and preschool children’s PA have been mixed [9,11,53,54]. Although we did not find an association between gross motor facilitating items and preschool-aged children’s average daily MVPA, this measure only accounted for the presence and accessibility of such items and not the amount of time the child played with each item or how the item was used given the lack of standardized observations of the child’s interactions with play equipment.

In exploratory analyses of other participant or household characteristics that may have contributed to variations in PA, we found that boys had 18 min more of daily MVPA than girls, which is approximately one-third of the overall daily recommendation of 60 min of MVPA for preschoolers [36]. The present finding in a home setting corroborated gender differences observed in young children in preschool environments [45] and in dense urban environments [49]. Gender differentials in PA were previously observed among adolescents in Dhaka [15], but the present data suggest that this gender gap in PA may start as early as 3 years of age, such that girls may accumulate less time spent active over their entire childhood.

The strengths of this study included high accelerometer wear compliance and objective measurement of PA and available indoor area. All participants had at least 10 h of daily accelerometer wear time for at least 3 days (2 weekdays and 1 weekend day), and measures of PA and indoor area were obtained using accelerometers and a laser measuring device with high inter- and intra-rater reliability. The study was novel in providing evidence to suggest that static factors, such as the physical environment of the home, are not major PA risk factors for young children in Dhaka.

Several limitations of the study should be acknowledged. First, as with most accelerometry research, we were unable to record observational PA data, such as activity type and context. For example, children were reportedly often carried during the day, so it is plausible that activity data captured during periods in which a child was being carried by the caregiver masked the child’s independently low levels of PA, obscuring associations between factors in the home built environment and PA. Second, the derivation of PA and SED categories (i.e., SED, LPA, MVPA) from accelerometry counts was highly sensitive to cut-point definitions, a pervasive problem in the pediatric accelerometry literature that has been referred to as the cut-point conundrum [55]. Third, we encountered practical challenges in obtaining objective measures of household hazards, such as unfinished construction. We originally hypothesized that such hazards would further constrain PA because parents would restrict their children’s active play to the safest areas of the home, but local study personnel did not consistently agree on whether a particular feature (e.g., cement blocks with exposed rebars) should be considered unfinished or a normal building condition. Additionally, we did not collect ancillary data on environmental variables, such as weather (e.g., daily temperature and amount of precipitation) and air quality, which may have partly explained the substantial day-to-day variations in the children’s PA. Future studies should explore the associations between sibling and maternal PA and child PA, differences in types of activities and active behaviors between genders, as well as the effects of seasonality between collection periods. Lastly, the sample size of this study in a small geographic area limited the precision of effect estimates and is an important limitation when considering the generalizability of the results with respect to the broader population of children in Dhaka or elsewhere.

## 5. Conclusions

In conclusion, we found that preschool-aged children in Dhaka usually engaged in more than an hour of MVPA per day and that despite living in dense urban settings in which mothers widely perceived play outside of the home to be unsuitable for young children, several characteristics of the home built environment (i.e., indoor area, household hazards, availability of gross motor activity items) were not associated with MVPA levels. The demonstrated feasibility and acceptability of using accelerometers to measure PA and SED behavior in preschool-aged children in Dhaka, Bangladesh, highlighted the potential applications of this tool in future epidemiological studies and trials in this setting. These results indicate that the physical environment of a preschool-aged child’s own home may not have a major impact on the child’s PA levels and, therefore, that future studies should focus on other potential determinants of PA, including behavioral factors (e.g., gender roles within the household, family perceptions of PA) and community-level characteristics of the natural and built environments.

## Figures and Tables

**Table 1:** Characteristics of study participants, their mothers, and the home environment.

Characteristic	Total *n* = 65	Boys *n* = 37	Girls *n* = 28
**Preschooler**			
Age (months), median (min, max)	37 (36, 38)	37 (36, 38)	37 (36, 38)
Height-for-age z-score, mean ± SD ^a^	−1.48 ± 1.0	−1.52 ± 0.8	−1.42 ± 1.2
Weight-for-age z-score, mean ± SD ^a^	−1.16 ± 1.1	−1.11 ± 1.0	−1.21 ± 1.3
BMI-for-age z-score, mean ± SD ^a^	−0.27 ± 1.0	−0.15 ± 1.0	−0.42 ± 1.1
Body fat (%), mean ± SD ^b^	24 (2.2)	24 (1.7)	24 (2.8)
Waist circumference (cm), mean ± SD ^c^	47.2 (3.7)	47.4 (3.5)	47.0 (4.1)
Anemia (hemoglobin < 110 g/L), *n* (%) ^d^	23 (36)	16 (43)	7 (26)
Carried by caregiver indoors, *n* (%)			
Never	18 (28)	9 (24)	9 (32)
Sometimes	45 (69)	26 (70)	19 (68)
Most of the time	2 (3)	2 (5)	0 (0)
Carried by caregiver when outdoors, *n* (%)			
Never	8 (12)	4 (11)	4 (14)
Very little	9 (14)	4 (11)	5 (18)
Sometimes	35 (54)	21 (57)	14 (50)
For long periods of time	13 (20)	8 (22)	5 (18)
**Mother**			
Age (years), median (min, max)	29 (20, 43)	29 (21, 40)	29 (20, 43)
Married	65 (100)	37 (100)	28 (100)
Level of education, *n* (%)			
Primary incomplete	14 (22)	6 (16)	8 (29)
Primary complete	7 (11)	2 (5)	5 (18)
Secondary incomplete	32 (49)	19 (51)	13 (46)
Secondary complete or higher	12 (18)	10 (27)	2 (7)
Primary occupation, *n* (%)			
Homemaker	62 (95)	36 (97)	26 (93)
Other	3 (5)	1 (3)	2 (7)
BMI (kg/m^2^) category, *n* (%) ^e^			
Normal weight (18.5–24.9)	26 (41)	18 (49)	8 (30)
Overweight (25.0–29.9)	25 (40)	16 (43)	9 (35)
Obese (>30.0)	12 (19)	3 (8)	9 (35)
Body fat (%), mean ± SD ^e^	35 ± 4.0	35 ± 3.8	35 ± 4.2
Waist circumference (cm), mean ± SD ^e^	91.1 ± 9.0	90.1 ± 9.2	92.4 ± 8.5
**Maternal perceptions**			
Adequate indoor space for PA, *n* (%)	43 (66)	25 (68)	18 (64)
Comfortable allowing child to play outdoors, *n* (%)	14 (22)	7 (19)	7 (25)
Conditions preventing child from playing outdoors, *n* (%)			
Unsafe outdoor environment	53 (82)	33 (89)	20 (71)
Unclean outdoor environment	36 (55)	25 (68)	11 (39)
Inadequate outdoor space	28 (43)	15 (41)	13 (46)
Poor weather	2 (3)	2 (5)	0 (0)
Child has siblings or friends to play with indoors, *n* (%)	52 (80)	28 (76)	24 (86)
Frequency of active play with siblings or friends, *n* (%)			
Every day	51 (78)	26 (70)	25 (89)
1–4 times per week	6 (9)	4 (11)	2 (7)
Almost never	7 (11)	6 (16)	1 (4)
**Household**			
Food security status, *n* (%) ^f^			
Severely food insecure	11 (17)	5 (14)	6 (21)
Moderately food insecure	23 (35)	13 (35)	10 (36)
Food secure	31 (48)	19 (51)	12 (43)
Outdoor space available, *n* (%)	57 (88)	35 (86)	22 (89)
Indoor area of the home (m^2^), median (IQR)	18.2 (17.8)	23.1 (16.7)	16.1 (17.9)
Min, Max	4.9, 59.4	4.9, 59.4	5.9, 46.9
Household occupancy (m^2^/person), mean ± SD	3.96 ± 2.26	4.23 ± 1.74	3.6 ± 2.80
Household person density (person/m^2^), mean ± SD	0.33 ± 0.18	0.29 ± 0.16	0.39 ± 0.20
Open stairwell, *n* (%) ^g^	30 (54)	18 (54)	12 (54)
Presence of at least one play item in the home, *n* (%)	33 (51)	22 (59)	11 (39)
Types of gross motor activity facilitating items, *n* (%) ^h^			
Fixed equipment (e.g., swings, jungle gym)	3 (5)	3 (8)	0 (0)
Ball equipment (e.g., bats, balls, rackets)	20 (31)	13 (35)	7 (25)
Locomotor equipment (e.g., tricycle, self-propelled car)	20 (31)	16 (43)	4 (14)
Rocking equipment (e.g., rocking horse)	4 (6)	2 (5)	2 (7)

BMI, body mass index (kg/m^2^); SD, standard deviation. ^a^ *n* = 63 (boys: *n* = 36; girls: *n* = 27); one male and one female participant refused to provide height measures and thus the height-for-age and BMI-for-age z-scores could not be calculated. All z-scores were standardized using World Health Organization age- and sex-specific growth curves. ^b^ *n* = 63 (boys: *n* = 36; girls: *n* = 27); body fat percentages were not calculated for one boy and one girl. ^c^ *n* = 63 (boys: *n* = 35; girls: *n* = 28); waist circumferences were not calculated for two boys. ^d^ *n* = 64 (boys: *n* = 37; girls: *n* = 27); one girl refused hemoglobin collection. ^e^ *n* = 63 (boys: *n* = 37; girls: *n* = 26); two mothers were pregnant and were excluded from BMI calculations and did not participate in bioelectrical impedance analysis or waist circumference collection. ^f^ A 2-item questionnaire developed by Hager et al. (2010) was used. ^g^ *n* = 55 (boys: *n* = 33; girls: *n* = 22); ten homes did not have access to a staircase. ^h^ In the 33 homes with at least one play item in the home, 47 total items were identified; categories are based on an adapted version of the Environmental Policy Assessment and Observation (EPAO) in daycare centers, as well as the Affordances in the Home Environment for Motor Development (AHEMD) assessment tools.

**Table 2:** Time and proportion of time spent sedentary and in physical activity using Trost cut-points.

Participant Characteristic or Setting	Number of Observation Days	Sedentary Time		Light Physical Activity		Moderate to Vigorous Physical Activity		Total Physical Activity	
		Average Min/d ^a^	Average % Wear Time ^a^	Average Min/d ^a^	Average % Wear Time ^a^	Average Min/d ^a^	Average % Wear Time ^a^	Average Min/d ^a^	Average % Wear Time ^a^
**All (*n* = 65)**	448	421 ± 48	52 ± 5.5	301 ± 37	37 ± 4.1	82 ± 23	10 ± 2.8	382 ± 49	48 ± 5.5
Boys (*n* = 37)	249	414 ± 36	52 ± 4.8	295 ± 33	37 ± 3.5	90 ± 24	11 ± 2.8	385 ± 47	48 ± 4.8
Girls (*n* = 28)	199	429 ± 58	53 ± 6.2	306 ± 42	38 ± 4.9	71 ± 18	8.9 ± 2.3	377 ± 53	47 ± 6.3
Food secure (*n* = 31)	211	417 ± 52	52 ± 5.8	298 ± 36	37 ± 4.2	85 ± 28	11 ± 3.4	383 ± 49	48 ± 5.8
Food insecure (*n* = 34)	237	423 ± 42	53 ± 5.3	301 ± 39	37 ± 4.1	79 ± 19	9.9 ± 2.2	380 ± 49	47 ± 5.3
Weekdays (*n* = 65) ^b^	319	419 ± 47	52 ± 5.6	300 ± 39	37 ± 4.2	83 ± 25	10 ± 3.0	383 ± 52	48 ± 5.6
Weekend days (*n* = 65) ^c^	129	426 ± 65	53 ± 6.9	298 ± 44	37 ± 5.0	80 ± 26	10 ± 3.2	377 ± 60	47 ± 6.9
Inside the home (*n* = 55) ^d^	411	346 ± 66	54 ± 6.0	236 ± 45	36 ± 4.8	66 ± 20	10 ± 2.7	301 ± 57	46 ± 6.0
Outside the home (*n* = 55) ^d^	250	70 ± 67	51 ± 12	49 ± 41	38 ± 8.5	15 ± 15	11 ± 6.1	63 ± 54	49 ± 11.5

^a^ Data are expressed as the mean ± standard deviation. ^b^ Weekdays are Sunday through Thursday in Bangladesh. ^c^ Weekend days are Friday and Saturday in Bangladesh. ^d^ The wear period was based on parent report; daily recall phone calls were made to collect information on the time spent outside the home; 10 participants did not have any parent-reported outdoor wear time. Due to suspected error in reporting, they were excluded from this comparison.

**Table 3:** Associations between household factors and average minutes of moderate to vigorous physical activity (MVPA) per day.

Household Factor	Unadjusted				Adjusted			
	Sample Size (Number of Observation Days)	Change in Min/d of MVPA	95% CI	*p*-Value	Sample Size (Number of Observation Days)	Change in Min/d of MVPA	95% CI	*p*-Value
Indoor area, per 10 m^2 a^	65 (448)	4.71	(0.34, 9.09)	0.035 *	65 (448)	2.91	(−3.36, 9.18)	0.363
Presence of an open stairwell ^b^	65 (448)	−4.58	(−16.2, 7.03)	0.439	65 (448)	−5.76	(−16.3, 4.80)	0.285
Presence of gross motor facilitating items ^c^	65 (448)	10.3	(−0.826, 21.3)	0.070	65 (448)	4.12	(−6.37, 14.6)	0.441

MVPA, moderate to vigorous physical activity; CI, confidence interval. ^a^ The adjusted model included household density (m^2^/person living in home), asset index score, maternal and paternal education category, and food security status. ^b^ The adjusted model included indoor area (m^2^), household density (m^2^/person living in home), asset index score, maternal and paternal education category, and food security status. ^c^ The adjusted model included child sex, asset index score, maternal and paternal education category, and food security status.

## Data Availability

The datasets generated and analyzed during the current study are available from the corresponding author on reasonable request.

## Supplementary Materials

The following are available online at https://www.mdpi.com/1660-4601/18/7/3362/s1: Table S1. Accelerometry parameter reporting. Table S2. Published activity count cut-points developed for ActiGraph© accelerometers to calculate sedentary time and physical activity level categories in toddlers and preschool-aged children. Table S3. Associations between hemoglobin concentration, anemia and average minutes of moderate to vigorous physical activity (MVPA) per day. Table S4. Associations between average minutes of moderate to vigorous physical activity (MVPA) per day and household factors with parent-reported sick-day outliers removed. Table S5. Inter-rater reliability of study measures of 10 randomly selected households. Table S6. Intra-rater reliability of study measures. Table S7. Accelerometer wear time. Table S8. Duration of activity and average wear time ± standard deviation (SD) at various levels of physical activity intensity and sedentary behavior among preschool-aged children in Dhaka, Bangladesh, by age-appropriate activity cut-points for ActiGraph© accelerometers during waking hours. Table S9. Unadjusted and multivariable-adjusted differences in daily average moderate to vigorous physical activity (MVPA) of preschool-aged children in Dhaka, Bangladesh based on the presence of gross-motor facility items in the home and other covariates. Figure S1. Participant flow chart. Figure S2. Association between average daily minutes of moderate to vigorous physical activity (MVPA) and hemoglobin concentration (g/L) in Bangladeshi preschoolers (*n* = 64). Blue and red hollow circles represent the observations contributed by boys and girls, respectively. Blue and red fit lines represent the model predictions from the adjusted mixed linear models with random child-specific intercepts, separated by boys and girls, respectively. Figure S3. Between- and within-subject variation in daily observations of total physical activity levels (min/d). Each vertical bar spans the maximum and minimum amount of daily total physical activity (min/day) of a single participant. The horizontal blue lines in each of the bars represent the median daily total physical activity of each participant.
