# Supplementary material for: Physical Activity and the Home Environment of Pre-School-Aged Children in Urban Bangladesh
Source: Int J Environ Res Public Health. 2021 Mar 24;18(7):3362. doi: 10.3390/ijerph18073362 (PMC8036728; doi:10.3390/ijerph18073362)

Additional File S1

Supplementary Table S1. Accelerometry parameter reporting.

Accelerometry parameters	Current study (n=65)
Brand and model of accelerometer used	ActiGraph© GT3X; tri-axial device
Sampling frequency data was collected in	30 Hz; raw counts obtained
Epoch length data was analyzed in	15-s time sampling intervals (epoch)
Placement of accelerometer	Right hip, anterior axillary line
Number of participants who received accelerometer	65
Accelerometer distribution method	In person; proper placement of devices was instructed by field research assistants
Days of data collection	7-14 days, all waking hours [a]
Criteria for defining non-wear periods	≥20 min of consecutive zeros
Valid wear-time criteria	
Number of valid days	≥3 days (2 weekdays, 1 weekend day)
Number of valid minutes/day	≥10 hours (600 min) of wear-time
PA outcome of interest	Activity counts/d; time (min/d and % of wear time) spent sedentary, and in LPA, MVPA, and TPA
Cut-points used for analysis	Trost *et al* (2011)[b]
Statistical technique used for analysis	Mixed-linear model with a random child-specific intercept
Number of non-compliant participants; number of participants with malfunctioning accelerometers	0; no monitor malfunctions

[a] Participants wore devices for a minimum of 7 days, but could continue wearing them up to 14 days if sick days were reported; Sick days were removed in sensitivity analyses.

[b] Cut-points were validated in uni-axial monitors; therefore, only activity counts from the vertical axis (Axis 1) were used for analyses.

Supplementary Table S2. Published activity count cut-points developed for ActiGraph© accelerometers to calculate sedentary time and physical activity level categories in toddlers and preschool-aged children.

	Intensity of activity (Counts/15 seconds)			Calibration and validation studies	
	Sedentary	Light	Moderate/ Vigorous	Participants	Criterion Measures
Trost *et al* (2011) [a]	0-48	49-418	≥419	2.1 ± 0.4 years (n=22)	Children's Activity Rating Scale (CARS)
Pate *et al* (2006) [b]	0-199	200-419	≥420	3-5 years (n=29)	VO_2 (portable metabolic unit)
Sirard *et al* (2005) [c]	0-301	302-614	≥615	3 years (n=5)	Modification of CARS
Van Cauwenberge *et al* (2011) [d]	0-372	373-584	≥585	5.8 ± 0.4 years (n=18)	Modification of CARS

[a] Sedentary = 0-195 cpmin; Light = 196-1672 cpmin; Moderate/Vigorous = ≥1673 cpmin.
[b] Sedentary = 0-799 cpmin; Light = 800-1679 cpmin; Moderate/Vigorous = ≥1680 cpmin.
[c] Sedentary = 0-1207 cpmin; Light = 1208-2459 cpmin; Moderate/Vigorous = ≥2460 cpmin.
[d] Sedentary = 0-1488 cpmin; Light = 1489-2339 cpmin; Moderate/Vigorous = ≥2340 cpmin.

Supplementary Table S3. Associations between hemoglobin concentration, anemia and average minutes of moderate-to-vigorous physical activity (MVPA) per day.

	Unadjusted				Adjusted			
	Sample size (number of observation days)	Change in min/d of MVPA	95% CI	p-value	Sample size (number of observation days)	Change in min/d of MVPA	95% CI	p-value
Hemoglobin concentration, g/L [a]	64 (434)	0.008	(-0.395, 0.412)	0.968	63 (427)	0.159	(-0.218, 0.536)	0.410
Anemia [a]	64 (434)	-0.538	(-12.5, 11.5)	0.930	63 (427)	-5.48	(-16.8, 5.82)	0.342

MVPA, moderate-to-vigorous physical activity; CI, confidence interval.

[a] Adjusted model controlled for child gender, child height-for-age z-score (HAZ), asset index score, maternal and paternal education category, and food security status.

Supplementary Table S4. Associations between average minutes of moderate-to-vigorous physical activity (MVPA) per day and household factors with parent-reported sick-day outliers removed.

	Unadjusted				Adjusted			
	Sample size (number of observation days)	Change in min/d of MVPA	95% CI	p-value	Sample size (number of observation days)	Change in min/d of MVPA	95% CI	p-value
Indoor area, per 10 m^2 [a]	65 (441)	5.14	(0.790, 9.49)	0.021*	65 (441)	3.30	(-2.96, 9.57)	0.302
Presence of an open stairwell [b]	65 (441)	-3.49	(-15.1, 8.16)	0.557	65 (441)	-4.75	(-15.3, 5.85)	0.380
Presence of gross-motor facilitating items [c]	65 (441)	10.6	(-0.53, 21.6)	0.062	65 (441)	4.50	(-6.01, 15.0)	0.401
Hemoglobin concentration, g/L [d]	64 (434)	0.008	(-0.395, 0.412)	0.968	63 (427)	0.159	(-0.218, 0.536)	0.410
Anemia [d]	64 (434)	-0.538	(-12.5, 11.5)	0.930	63 (427)	-5.48	(-16.8, 5.82)	0.342

CI = Confidence interval

[a] Adjusted model controlled for household density (m^2/person living in home), asset index score, maternal and paternal education category, and food security status.

[b] Adjusted model controlled for indoor area (m^2), household density (m^2/person living in home), asset index score, maternal and paternal education category, and food security status.

[c] Adjusted model controlled for child gender, asset index score, maternal and paternal education category, and food security status.

[d] Adjusted model controlled for child gender, child height-for-age z-score (HAZ), asset index score, maternal and paternal education category, and food security status.

Supplementary Table S5. Inter-rater reliability of study measures of 10 randomly selected households.

	Pearson's Correlation coefficient	Absolute difference between measures, Mean (min, max)
Child measures		
Waist circumference, cm	0.9838	0.385 (0.100, 0.950)
Resistance, ohms	0.9916	12.1 (1.00, 29.0)
Reactance, ohms	0.9285	2.30 (0, 6.0)
Body fat, %	0.9706	0.269 (0.019, 0.647)
Maternal measures		
Waist circumference, cm	0.9985	0.420 (0.050, 0.950)
Resistance, ohms	0.9405	19.2 (1.50, 50.0)
Reactance, ohms	0.9958	0.650 (0, 2.0)
Body fat, %	0.9845	0.866 (0.054, 2.03)
Household measures		
Home area, m^2	0.999	0.449 (0.011, 1.22)

Supplementary Table S6. Intra-rater reliability of study measures.

	Number of observations	Pearson's correlation coefficient for paired measures	Absolute difference between paired measures, Mean (min, max)
Child measures			
Height, cm	63	0.999	0.137 (0, 0.50)
Weight, kg	65	0.997	0.054 (0, 1.15)
Waist circumference, cm	63	0.998	0.211 (0, 0.50)
Resistance, ohms	63	0.999	1.63 (0, 8.0)
Reactance, ohms	63	0.990	1.20 (0, 7.0)
Maternal measures			
Height, cm	65	0.999	0.062 (0, 0.20)
Weight, kg	63	1.000	0.052 (0, 0.20)
Waist circumference, cm	63	0.999	0.229 (0, 1.70)
Resistance, ohms	63	0.999	0.556 (0, 4.0)
Reactance, ohms	63	0.997	0.270 (0, 3.0)
Household measures			
Length, m*	65	0.998	0.01 (0, 0.44)

* The average correlation coefficient between duplicate measurements of two length measures (length A and length B) across all homes.

Supplementary Table S7. Accelerometer wear time.

	Total (n=65)	Boys (n=37)	Girls (n=28)
Number of valid days of accelerometry			
Median (IQR)	7 (0)	7 (1)	7 (0)
Min, Max	3, 10	3, 8	6, 10
Average wear time, hours per valid day			
Median (IQR)	13.6 (1.0)	13.5 (0.9)	13.7 (1.1)
Min, Max	11.2, 14.3	11.8, 14.2	11.2, 14.3
Number of valid week day observations [a]			
Median (IQR)	5 (0)	5 (1)	5 (0)
Min, Max	2, 6	2, 6	4, 6
Number of valid weekend day observations [b]			
Median (IQR)	2 (0)	2 (0)	2 (0)
Min, Max	1, 4	1, 2	2, 4

IQR = Interquartile range.

[a] Week days are Sunday through Thursday in Bangladesh.

[b] Weekend days are Friday and Saturday in Bangladesh.

Supplementary Table S8. Duration of activity and average wear time ± standard deviation (SD) at various levels of physical activity intensity and sedentary behavior among preschool-aged children in Dhaka, Bangladesh, by age-appropriate activity cut-points for ActiGraph© accelerometers during waking hours.

Cut-points	Sedentary time		Light physical activity		Moderate-to-vigorous physical activity		Total physical activity	
	Average min/d mean ± SD	Average % wear time, mean ± SD	Average min/d mean ± SD	Average % wear time, mean ± SD	Average min/d mean ± SD	Average % wear time, mean ± SD	Average min/d mean ± SD	Average % wear time, mean ± SD
Trost et al (2011)	421 ± 48	52 ± 5.5	301 ± 37	37 ± 4.1	82 ± 23	10 ± 2.8	382 ± 49	48 ± 5.5
Pate et al (2006)	609 ± 46	76 ± 4.5	112 ± 19	14 ± 2.2	82 ± 23	10 ± 2.9	193 ± 38	24 ± 4.5
Sirard et al (2005)	672 ± 43	84 ± 3.7	93 ± 19	12 ± 2.3	37 ± 14	4.6 ± 1.7	130 ± 31	16 ± 3.7
Van Cauwenberge et al (2011)	704 ± 42	88 ± 3.1	57 ± 12	7.1 ± 1.5	42 ± 15	5.2 ± 1.8	98 ± 26	12 ± 3.1

* Wear time parameters: participants wore ActiGraph© GT3X accelerometers on their right hip for a minimum of 3 days with a minimum of 10 hours of valid wear time; non-wear periods are defined as having 20 minutes or more of consecutive zeros in the vertical axis.

Supplementary Table S9. Unadjusted and multivariable-adjusted differences in daily average moderate-to-vigorous physical activity (MVPA) of preschool-aged children in Dhaka, Bangladesh based on the presence of gross-motor facility items in the home and other covariates.

	Unadjusted			Adjusted [a]		
	Change in min/d MVPA	95% CI	p-value	Change in min/d MVPA	95% CI	p-value
Presence of gross-motor activity facilitating items in the home						
Not present	-	-	-	-	-	-
At least one is present	10.3	(-0.83, 21.3)	0.070	4.12	(-6.37, 14.6)	0.441
Gender						
Boys	-	-	-	-	-	-
Girls	-18.7	(-29.3, -8.24)	<0.001***	-14.6	(-25.0, -4.09)	0.006**
Asset index score	4.37	(1.08, 7.65)	0.009**	2.55	(-1.24, 6.35)	0.187
Maternal education						
Secondary complete or higher	-	-	-	-	-	-
Secondary incomplete	-22.1	(-36.3, -7.91)	0.002**	-19.0	(-33.6, -4.49)	0.010*
Primary complete	-27.3	(-47.2, -7.36)	0.007**	-17.3	(-38.1, 3.45)	0.102
Primary incomplete	-24.7	(-41.2, -8.23)	0.003**	-16.4	(-33.4, 0.61)	0.059
Paternal education						
Secondary complete or more	-	-	-	-	-	-
Secondary incomplete	5.78	(-9.16, 20.7)	0.448	9.33	(-4.35, 23.0)	0.181
Primary complete	-2.67	(-21.2, 15.8)	0.777	7.09	(-9.62, 23.8)	0.406
Primary incomplete	-5.87	(-23.9, 12.1)	0.522	12.4	(-4.75, 29.5)	0.157
No schooling	-10.2	(-33.4, 13.0)	0.389	8.15	(-14.2, 30.5)	0.475
Food security status						
Food insecure	-	-	-	-	-	-
Food secure	5.65	(-5.63, 16.9)	0.326	-1.00	(-11.2, 9.16)	0.847

[a] Adjusted model included presence of gross-motor activity facilitating items in the home, child gender, asset index score, maternal and paternal education category, and food security status.

* p-value ≤ 0.1; statistically significant

** p-value ≤ 0.01; statistically significant
*** p-value ≤ 0.001; statistically significant

Supplementary Figure S1. Participant flow chart.

184 MDIG participants
(from PTC) contacted

58 could not be successfully
contacted (after 3 attempts)

126 individuals successfully contacted

39 were not interested

87 interested individuals

17 were not eligible

9 had moved out of catchment area
6 did not have access to a mobile phone
2 had a congenital anomaly

Congenital Anomalies:
1 clubfoot which caused pain
1 diagnosed with heart
disease after 2 years of age

70 eligible individuals

5 refused prior to
scheduling home visit

2 fathers refused their child's participation
2 mothers did not think that their child would cooperate
1 could not find time to schedule home visit

Number of individuals enrolled
(n=65)

Supplementary Figure S2. Association between average daily minutes of moderate-to-vigorous physical activity (MVPA) and hemoglobin concentration (g/L) in Bangladeshi preschoolers (n=64). Blue and red hollow circles represent the observations contributed by boys and girls, respectively. Blue and red fit lines represent the model predictions from the adjusted mixed linear models with random child-specific intercepts, separated by boys and girls, respectively.

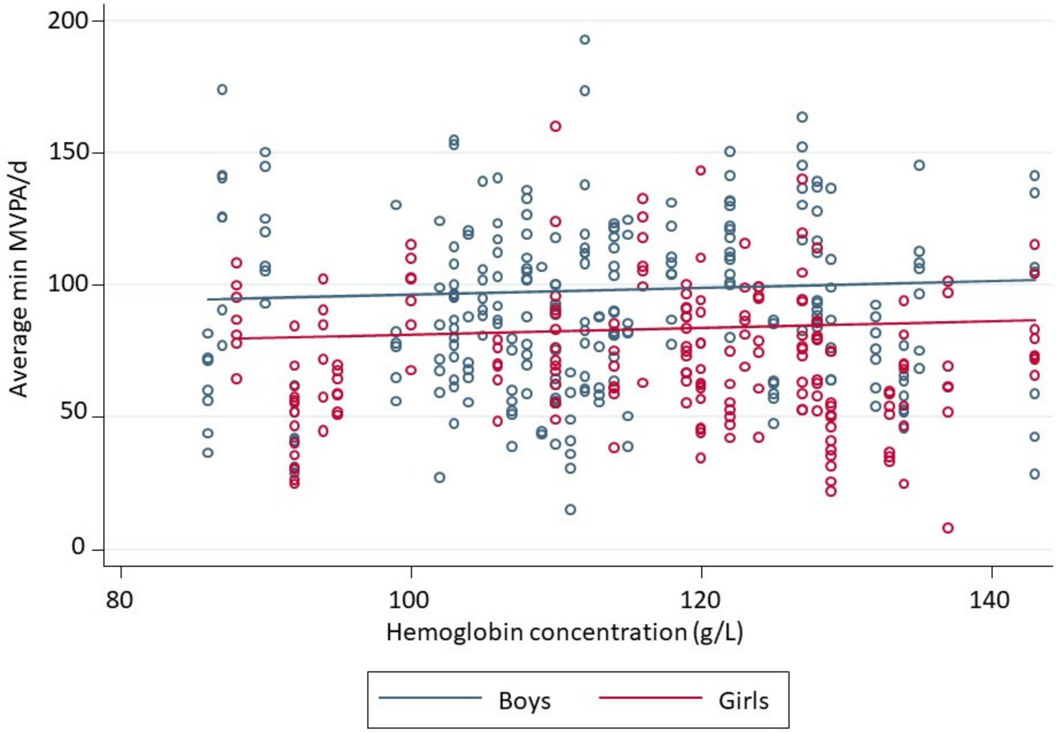

Supplementary Figure S3. Between- and within-subject variation in daily observations of total physical activity levels (min/d). Each vertical bar spans the maximum and minimum amount of daily total physical activity (min/day) of a single participant. The horizontal blue lines in each of the bars represent the median daily total physical activity of each participant.

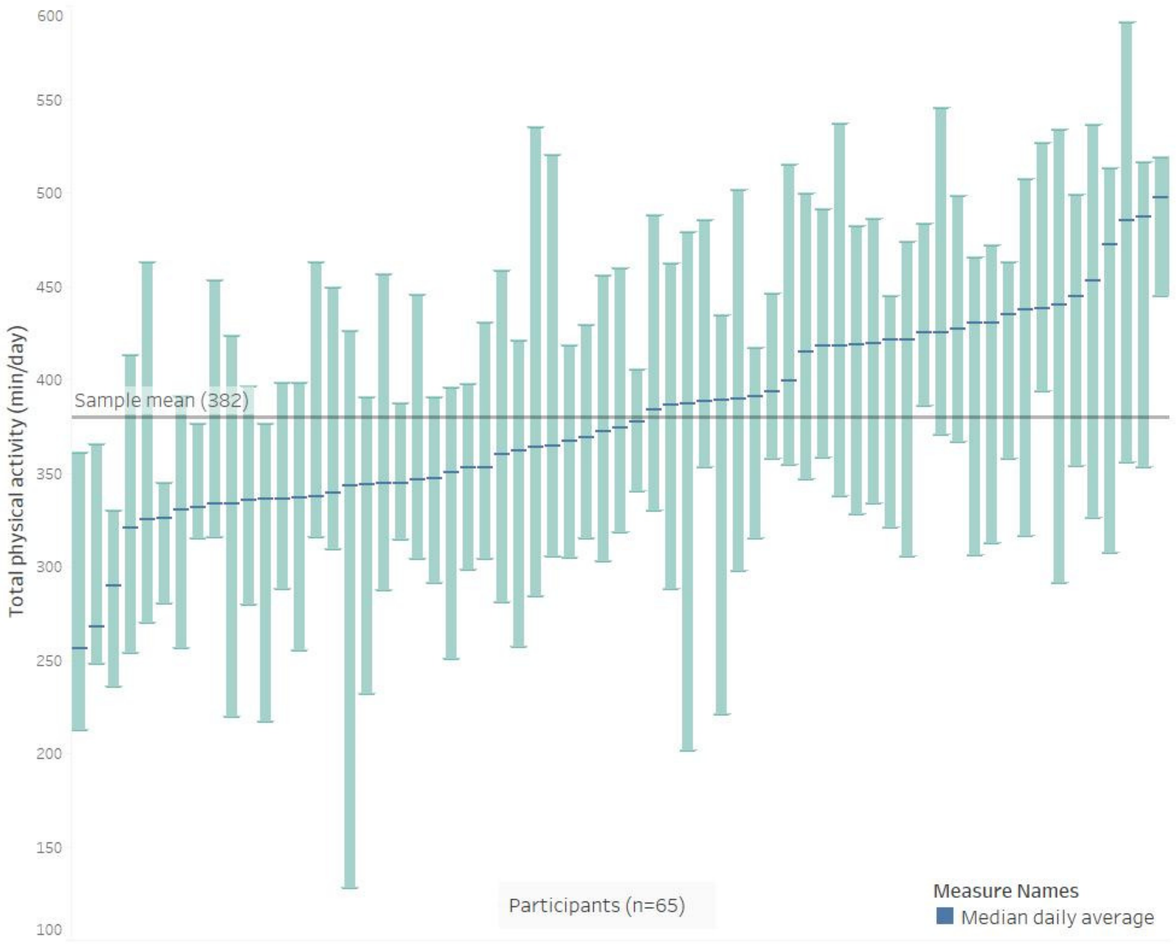

Supplement: Supplementary file 1 [file ijerph-18-03362-s001.pdf]
